# Development of Carbon-Nanodot-Loaded PLA Nanofibers and Study of Their Barrier Performance for Medical Applications

**DOI:** 10.3390/nano13071195

**Published:** 2023-03-27

**Authors:** Muhammad Usman Munir, Thomas Mayer-Gall, Jochen S. Gutmann, Wael Ali, Omid Etemad-Parishanzadeh, Haleema Khanzada, Daiva Mikučioniene

**Affiliations:** 1 Department of Production Engineering, Faculty of Mechanical Engineering and Design, Kaunas University of Technology, Studentų 56, LT-51424 Kaunas, Lithuania; haleema.khanzada@ktu.edu (H.K.); daiva.mikucioniene@ktu.lt (D.M.); 2 Deutsches Textilforschungszentrum Nord-West gGmbH, Adlerstr. 1, D-47798 Krefeld, Germany; mayer-gall@dtnw.de (T.M.-G.); ali@dtnw.de (W.A.); omidetemad@dtnw.de (O.E.-P.); 3 Institute of Physical Chemistry, Center for Nanointegration (CENIDE), University of Duisburg-Essen, Duisburg-Essen, Universitätsstraße 2, D-45117 Essen, Germany

**Keywords:** electrospinning, nanofibers, sustainability, poly lactic acid, antimicrobial activity, carbon nanodots, personal protective equipment

## Abstract

The COVID-19 pandemic has increased the usage of personal protective equipment (PPE) all round the world and, in turn, it has also increased the waste caused by disposable PPE. This has exerted a severe environmental impact, so in our work, we propose the utilization of a sustainable electrospun nanofiber based on poly lactic acid (PLA), as it is biobased and conditionally degradable. We optimized the weight percentage of the PLA-precursor solution and found that 19% PLA produces fine nanofibers with good morphology. We also introduced carbon nanodots (CNDs) in the nanofibers and evaluated their antibacterial efficiency. We used 1, 2, 3, and 4% CNDs with 19% PLA and found increased antibacterial activity with increased concentrations of CNDs. Additionally, we also applied a spunbond-nanofiber layered assembly for the medical face masks and found that with the addition of only 0.45 mg/cm^2^ on the nonwoven sheet, excellent particle filtration efficiency of 96.5% and a differential pressure of 39 Pa/cm^2^ were achieved, meeting the basic requirements for Type I medical face masks (ASTM-F2100).

## 1. Introduction

The sudden outbreak and rapid transmission of the SARS-CoV-2 (COVID-19) virus has encouraged people across the globe to adopt safety measures to help stop the spread of this infectious disease. Thus, there has also been a sudden increase in the demand for PPE, such as face masks and gowns. Globally, the market size of PPE in 2022 was USD 80 billion, and this was projected to rise to USD 111 billion by 2029. Furthermore, due to COVID-19, the market size for PPE was increased by 17% in 2020 compared to 2019 [1,2]. Severe acute respiratory syndrome coronavirus 2 (COVID-19) is a contagious disease that mainly attacks the respiratory system of the patient [3]. Its first case was reported in December 2019, and its origin was related to food markets [4]. Due to this outbreak, on 11 March 2020, the WHO declared the COVID-19 virus a pandemic [5].

The transmission of COVID-19 is mainly caused by aerosols exhaled by a COVID-positive individual to a by-stander within a range of 1 m. People infected with COVID-19 can spread the virus in the shape of respiratory droplets 5 microns in size when they exhale, cough, sneeze, speak, or sing. These exhaled respiratory droplets (containing the COVID-19 virus) can be inhaled by other people directly and can also be transmitted if the droplets touch the eyes of other people [6]. Another reason for the spread of COVID-19 is the poor ventilation systems in crowded indoor areas, as air contaminated with the virus is not refreshed, and people present are likely to contract the virus. This is also the reason for the decrease in COVID-19-positive cases in summer compared to the winter season, as in summer, doors and windows are kept open for ventilation (making the air fresh) and in winter, windows and doors are kept closed to mitigate the cold weather, increasing the spread of COVID-19 due to poor ventilation [7]. The only way to stop the airborne spread of respiratory droplets is by using a face mask and keeping physical distance between people. Medical face masks are very effective against the spread, as they have good barrier performance and are also breathable.

As the sudden outbreak has increased the usage of disposable medical face masks, globally, this has generated an estimated waste of 7200 tons per day [8]. Generally, medical face masks and gowns are made from nonwoven polypropylene sheets or other man-made fibers/polymers. These polymers are not usually biodegradable; hence, they impart significant risks to land and aquatic life. According to United Nations’ environment program, 75% of used face masks eventually enter landfill or float in the sea [2]. Saliu et al. [9] studied the releases of microplastics in artificial weathering experiments. They found that a single surgical mask submitted to 180 h of UV-light irradiation and vigorous stirring in artificial seawater may release up to 173,000 microplastic fibers/day. This release of microplastics is potentially dangerous to both marine flora and humans. To overcome this pollution challenge, many researchers have shifted their research work to the development of medical face masks with biodegradable and sustainability features. Munir et al. [10] proposed a composite of poly vinyl alcohol (PVA)/Aloe vera/ZnO nanowebs for potential use in medical devices. They found that these composite nanowebs also have antimicrobial properties as they are also composed of Aloe vera and ZnO nanoparticles (NPs). This composite assembly is more sustainable due to the hydrophilic and water-dissolution properties of PVA. Das et al. [11] proposed a face mask composed of a wheat-gluten biopolymer. This can be spun into nanofibers and subsequently carbonized at over 700 °C to form a network structure, which can simultaneously act as the filter medium and as a reinforcement for gluten-based masks. Tiliket et al. [12] developed a cellulose-based sheet through the chemical modification of the surface of low-cost non-woven cellulosic fiber filters, followed by the fixing of poly(ethylenimine) (PEI) to give them an antiviral property. They found the best possible results with two layers of cellulosic fiber materials functionalized with PEI. Patil et al. [13] developed a biodegradable three-ply face mask containing cotton-PLA-cotton layers. The inner nanofiber PLA layer was encapsulated with phytochemicals and was electrospun on the cotton layers by needle-based electrospinning. They demonstrated an enhanced differential pressure of 35.78 Pa/cm^2^ and found that the bacterial filtration efficiency was above 97.9%. Choi et al. [14] developed hydrophobic, highly breathable, and high-performance mask filter by integrating two biodegradable microfiber and nanofiber mats into a Janus membrane filter and then coating it with cation-charged chitosan nanowhiskers. They reported that the formed filter provided lower differential pressure, which is suitable for breathing. Wang et al. [15] worked on developing a PLA/titania fibrous membrane and reported that with only 1.75% titania, the membrane exhibited 99.5% antimicrobial activity. In addition, a high particle filtration of 99.996% and a low pressure drop of 128.7 Pa were also reported. He et al. [16] printed PLA on a PLA-nanofiber sheet to fabricate a nano-porous structure with a transparent appearance. They claimed that the transparent appearance overcomes the threatening appearance of masks, which can be a feasible way of reducing the social trauma caused by COVID-19 pandemic. Kadam et al. [17] developed biodegradable nanofiber mats containing gelatin/β-cyclodextrin. They reported that these nanofiber membranes captured aerosols (0.3–5 μm) with a filtration efficiency of more than 95%. Al Azeem et al. [18] proposed a sustainable medical face mask from biopolymer polyhydroxyalkanoates (PHAs). This polymer is biodegradable and has good porosity, making it suitable for use in medical face masks.

Whether by using antimicrobial polymers or by adding antimicrobial agents, researchers have opted to impart antimicrobial properties to the polymers used for medical face masks. In our previous study, we also studied the comparative antimicrobial properties of Aloe vera and ZnO NPs in PVA nanowebs. We found that the ZnO NPs offered superior antimicrobial activity to Aloe vera, which was entirely due to the photocatalysis property of ZnO NPs. Among the emerging material with antimicrobial properties are carbon nanodots (CNDs), or carbon quantum dots [19]. These CNDs have photocatalytic properties resembling semiconductor nanoparticles. The main advantage of using CNDs rather than semiconductor nanomaterials is their visible-light photocatalytic properties. Furthermore, CNDs have broad and strong absorption in the visible and in the near IR region. According to recent research, the antimicrobial activity of CNDs is associated with the generation of reactive oxygen species (ROS). When the incident light hits CNDs, it generates ROS by activating the oxygen in air or water, leading to the formation of hydroxyl free radicals (·OH) or single oxygen atoms. These generated ROS can destroy the biomolecules/cells of bacteria in contact with CNDs [19]. Meziani et al. [20] first reported that CNDs under visible/natural light can effectively inhibit *E. coli* cells both in suspensions and on the agar surface.

To address the current environmental impact and disposal-related issues with medical face masks, we propose to develop a nanofiber sheet or web to replace the inner layer in medical face masks. The inner layer in medical face masks is composed of a melt-blown nonwoven web, and it essentially serves as a filtration layer, sandwiched between two layers of spunbonded nonwoven sheets. We also propose that the material cost is reduced to almost 75% with the nanofiber layer compared to the meltblown layer. Furthermore, we introduce CNDs as emerging photoactive materials in PLA nanowebs, having found that they offer good antimicrobial properties against *S. aureus* and *E. coli*. To our knowledge, this work has not yet been reported.

## 2. Materials and Methods

### 2.1. Materials

The biopolymer polylactic acid 4060D, in pellet form, was purchased from NatureWorks LLC. It has a melting temperature of 210 °C. Dimethyl formamide was purchased from Merck. Glucosamine hydrochloride (98% purity) from Thermos Fischer, Shanghai, China and 1,3-diaminobenzene (99% purity) was purchased from Carl Roth.

### 2.2. Synthesis of CNDs

For the synthesis of CNDs, glucosamine hydrochloride (1.00 g, 4.63 mmol) was dissolved in deionized H_2_O (20 mL) and agitated to achieve complete dissolution in 250-mL Erlenmeyer conical flask. Next, 1,3-diaminobenzene (0.55 g, 5.10 mmol) dissolved by sonication in methanol (10 mL) was added to the glucosamine solution and further agitated to obtain solution homogeneity. The conical flask was then placed in microwave, in a well-ventilated area, and then irradiated for 3 min (800 W, 90% power). A viscous brown oil-like material was produced, which was dissolved in water (10 mL) and centrifuged at 4000 rpm for 1 h. Next, the bulk solution was reduced in vacuo to obtain brown powder. The CNDs were then dispersed in DMF and used in different weight percentages following the design of experiment defined in Table 1.

### 2.3. Preparation of Solution for Electrospinning

**Figure 1:**
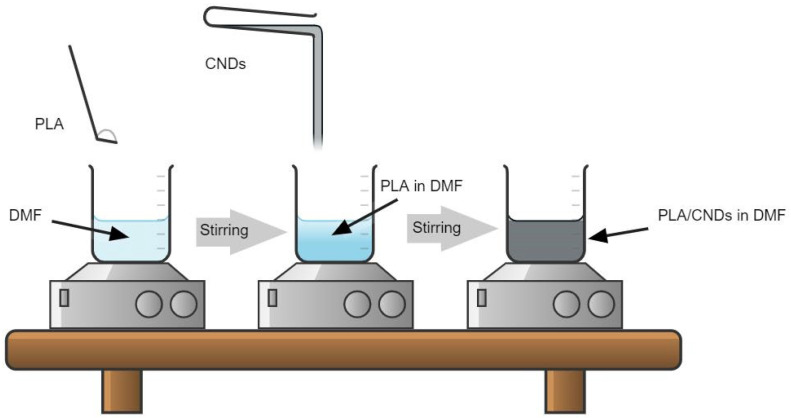
Preparation of solution for electrospinning.

The PLA was first dissolved in DMF in desired quantity and then stirred for about 6 h to produce a homogenous solution. Figure 1 shows a schematic diagram for the preparation of electrospinning solution. All samples were stirred at 300 rpm at room temperature. The 12, 15, 17 and 19% PLA in DMF were prepared, followed by their electrospinning and characterization. After evaluating SEM images of all samples, we selected the weight percentage with best morphology and then used it to evaluate barrier performance with variations in electrospinning time (weight add-on).

### 2.4. Electrospinning of PLA

**Figure 2:**
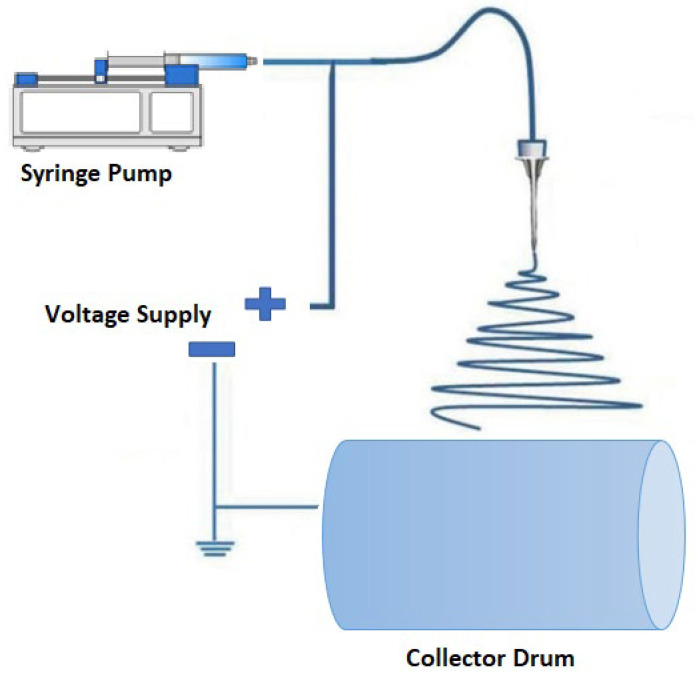
Electrospinning process for PLA nanofibers.

The nanofiber sheets were prepared by electrospinning technique. All solutions with varying amounts of PLA and CNDs are presented in Table 1. The electrospinning machine was self-made, a schematic diagram of machine is shown in Figure 2, featuring a needle pump, a voltage device, and a collector drum. The area of rotating-drum collector was 255 cm^2^ and the feed rate was maintained through a syringe pump at 0.4 mL/h. The machine was enclosed in a see-through container and temperature/humidity were maintained automatically by a device. The needle was placed vertically to that of collector drum with a maintained distance of 17 cm. A potential difference of 23 KV was applied for the generation of PLA nanofibers. 

### 2.5. Characterization

The rheological properties, such as viscosity of PLA solutions, were measured by Viscometer B-ONE PLUS (LAMY RHEOLOGY Instruments). First, a zero setting was measured by removing the spindle, after which viscosity of PLA solution was measured with spindle R-6, at 600 rpm, for 60 s. The electrical conductivity of PLA solutions was determined with conductivity meter (TDS & EC meter, PATEA). The surface morphology of nanofibers was observed with scanning-electron microscope (SEM), (Hitachi Model S-3400N, Hitachi High Technologies Europe GmbH, Tokyo, Japan). To check the fiber diameter, we analyzed the SEM images with ImageJ software by taking an average of thirty readings from each sample. Dynamic light scattering (DLS) was performed to determine the size distribution of the CNDs using a Zetasizer 1000 apparatus (Malvern Panalytical Ltd., Malvern, UK). The sample was diluted to minimize the agglomeration. Measurements were conducted at an angle of 90° and a temperature of 25 °C. To check the chemical composition of the formed nanofibers, we subjected them to FTIR (Shimadzu IRPrestige21) spectroscopy. Thermogravimetric analysis (TGA) (TA Instrument TGA55) was performed to check any residual DMF in the nanofibers. The aluminum oxide crucible was used to perform TGA with a heating rate of 10 °C/min.

## 3. Performance Testing

### 3.1. Antimicrobial Properties

The antimicrobial activity was analyzed by plate-count method (quantitative measurement), defined in ISO 20645:2004 standard. The PLA/CND electrospun nanofibers were tested against Gram-negative bacteria (*E. coli*) and Gram-positive bacteria (*S. aureus*). The test specimens were taken in a specific grammage and then placed in the vial. As the samples were hydrophobic, they were placed in the bacterial solution with the help of glass rod. All vials were shaken well on a mixer to obtain homogenous solution. All vials were then incubated with a D-65 light source at 37 ± 2 °C for 18 to 24 h. After incubation, the antimicrobial activity was calculated according to ISO 20645: 2005 Annex C: Quantitative measurement by plate-count method. The antimicrobial activity was measured against the control vial with pure PLA nanofibers.

### 3.2. Particulate Filtration Efficiency (PFE) Test

The submicron-particulate-filtration efficiency of prepared samples was analyzed by particulate-efficiency tester from Lorenz Meßgerätebau GmbH & Co. KG, Katlenburg-Lindau, Germany, which determined the filtration efficiency to particles of latex spheres/NaCl. We used NaCl aerosols for the PFE test. To carry out this test, ASTM F2299 was followed. The PFE tester measures filtration efficiency by comparing the particle count in the feed stream (upstream) to that in the filtrate (downstream). The PFE utilizes filtered and dry air through an automizer, which is used to produce aerosol containing latex spheres with particle sizes in range of 0.1 to 5.0 μm and airflow-test velocities of 0.5 to 25 cm/s. Each sample was clamped into the fixtures and then mounted on the machine, and the test procedure was initiated. The generated aerosol was passed through the sample material, and the photometer detected the upstream and downstream counts of aerosol particles and generated a report on percentage filtration efficiency for different sizes of aerosol.

### 3.3. Differential Pressure

Air permeability of prepared samples was determined by a differential-pressure tester from Qinsun Instruments Co., Ltd., (Hong Kong, China) model G285, with test head of 4.9 cm^2^ and differential-pressure-sensor range from 50 to 500 Pascals. Following EN 14683 test method, air was drawn at the rate of 8 L/min to a measured area (25 mm in diameter, or 4.9 cm^2^) of the sample, and the differential pressure was measured in Pa/cm^2^.

## 4. Results

### 4.1. SEM Analysis

**Figure 3:**
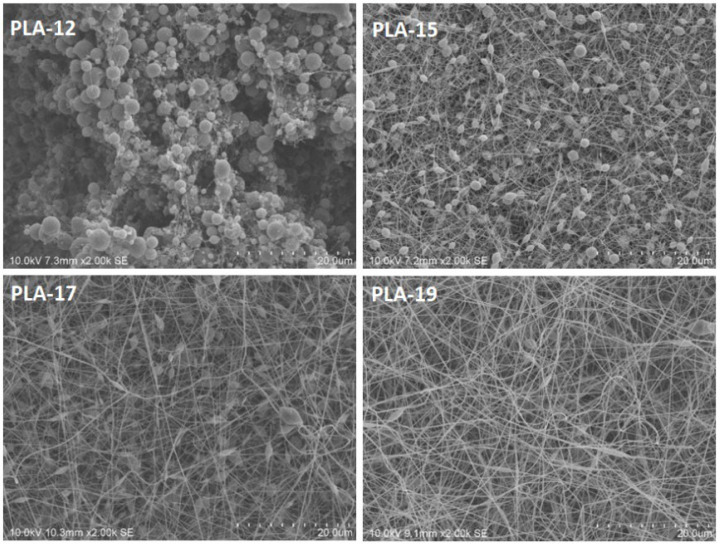
SEM images of pure PLA nanofibers with concentrations of 12, 15, 17, and 19% (scale—20 µm).

The SEM analysis described the morphology of the as-formed nanofibers on the nonwoven sheets. With the help of ImageJ software, we measured the average diameter of each sample. About thirty measurements were taken on each SEM image on the same fiber at different locations and on different fibers, and then the average diameter of the nanofibers was calculated. One of the most important parameters that influences the uniform formation of electrospun nanofibers, and their diameter is the concentration of the precursor solution. We started with 12% pure PLAs in DMF (without CNDs) to check their spinnability, and we observed that rather than forming nanofibers, the droplets were collected on the drum collector. This was because low concentrations provide low viscosity, which favors droplet formation rather than nanofiber formation [21]. Figure 3 shows that when we increased the concentration of PLA to 15%, 17%, and 19%, and the agglomeration decreased, and smooth nanofibers were formed [22]. This phenomenon was also observed by Fong et al. [23]. Higher viscosity favors the formation of fibers without beads, and higher net charge density not only favors the formation of fibers without beads, but also favors the formation of thinner fibers.

**Figure 4:**
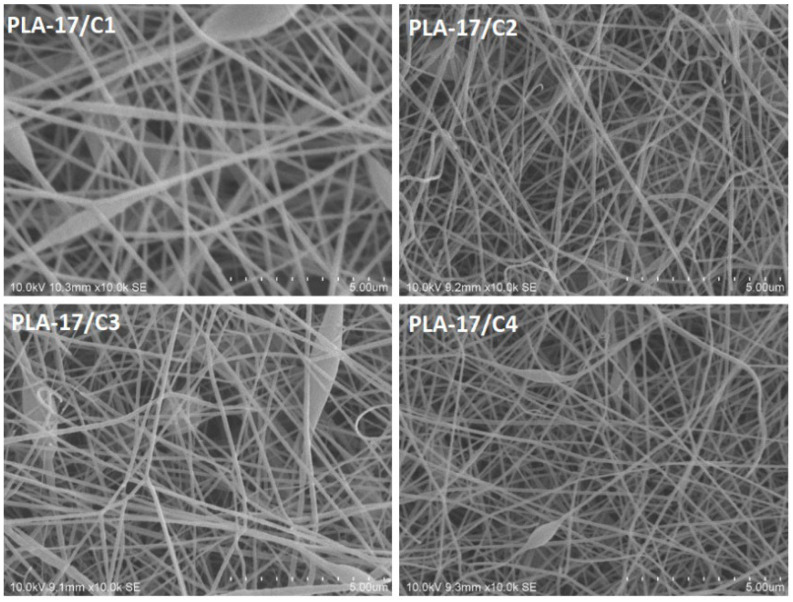
SEM images of 17% PLA nanofibers with variations in the CNDs of 1, 2, 3, and 4% (scale—5 µm).

For the electrospinning of the PLA/CNDs, it was observed that with the increase in the concentration of conductive CNDs, the obtained average diameter of the nanofibers decreased, as shown in Figure 4. For instance, the average diameter obtained for the PLA-17/C1 nanofibers was around 178 ± 37 nm, while that obtained for the PLA-17/C4 was 98 ± 25 nm. The increase in the concentration of CNDs decreased the diameters of the nanofibers, since CNDs are highly conductive, and conductivity results in finer and smoother nanofibers, as observed by other researchers [23,24].

**Figure 5:**
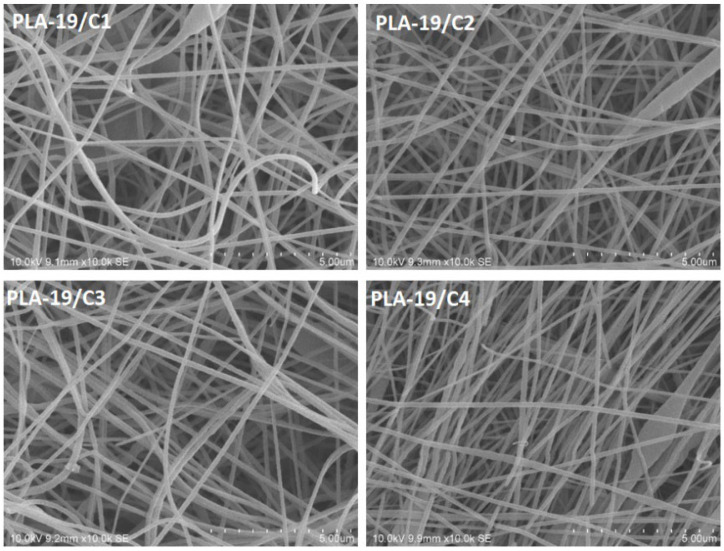
SEM images of 19% PLA nanofibers with variations in CNDs of 1, 2, 3, and 4% (scale—5 µm).

Similar behavior was also observed in the samples with 19% PLA with varying concentrations of CNDs, as shown in Figure 5. The average diameter of the nanofibers decreased from 150 ± 40 nm (PLA-19/C1) to 88 ± 25 nm (PLA-19/C4) when the concentration of CNDs increased from 1 to 4%.

### 4.2. Size Analysis of CNDs

**Figure 6:**
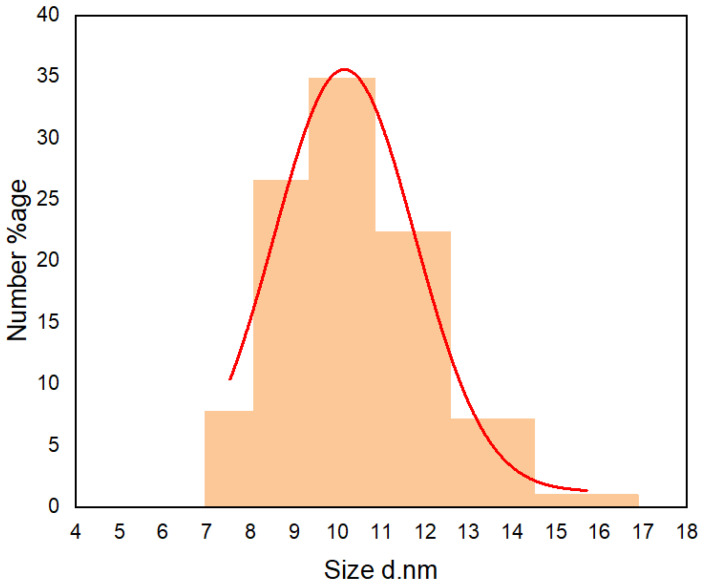
Size distribution of prepared CNDs measured by DLS.

The hydrodynamic size analysis was conducted on a zeta sizer (DLS). Figure 6 shows the size distribution of the CNDs. The CND particle sizes were between 7.5 nm and 15.7 nm, with 10.2 nm as the mean size. Furthermore, almost 92% of the particles had a size between 7.5 nm and 11.7 nm. We did not observe the physical presence of CNDs in any of the SEM images of the formed nanofibers; this was due to the very small size of the CNDs and the comparatively large size of the nanofibers (above 100 nm). These nanodots were embedded in the fibers and on surface; thus, they could not be seen as an agglomeration in the SEM images.

### 4.3. Antimicrobial Testing

**Figure 7:**
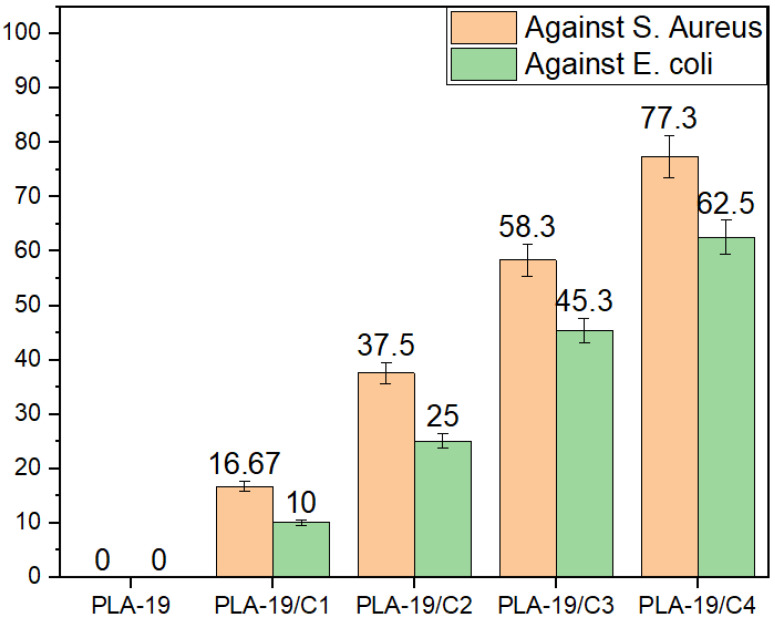
Antimicrobial activity of PLA-19-C1, PLA-19/C2, PLA-19/C3 and PLA-19/C4 against *S. aureus* and *E. coli*.

Amongst all the weight percentages of the PLA in the DMF, we found that 19% PLA produced fine nanofibers. Therefore, we tested the antimicrobial activity of the PLA-19 with different concentrations of CNDs. Figure 7 shows that the pure PLA nanofibers exhibited no antimicrobial activity against either the *S. aureus* or the *E. coli*. In contrast, the integration of the CNDs resulted in an antimicrobial activity and with the increase in the concentration of the CNDs in the PLA nanofibers, the antimicrobial activity also increased.

**Figure 8:**
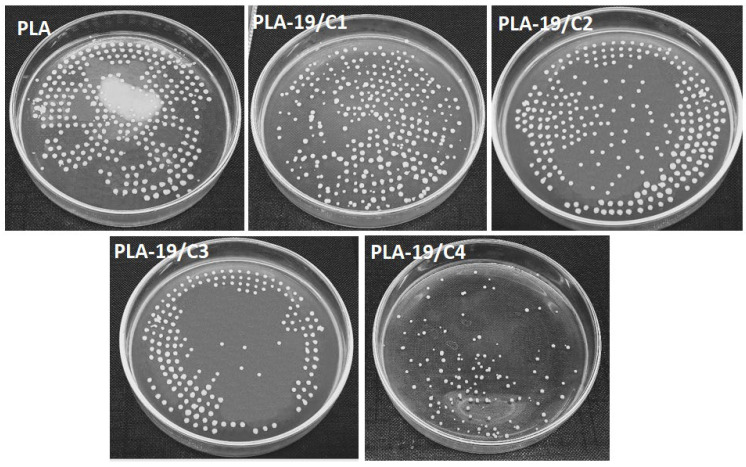
Quantitative measurement of antimicrobial activities of 19% PLA nanofibers with different CND concentrations against *E. coli* bacteria.

The quantitative measurements of the antimicrobial activity against *E. coli* are shown in Figure 8. For the antimicrobial activity against the *E. coli*, PLA-19/C1 exhibited only 10% antimicrobial efficiency, which increased to 62.5% with the 4% CNDs. Since the CNDs were also positioned on the surfaces of the PLA nanofibers, more sites for reactive oxygen species were present and, in turn, more microbes were killed.

**Figure 9:**
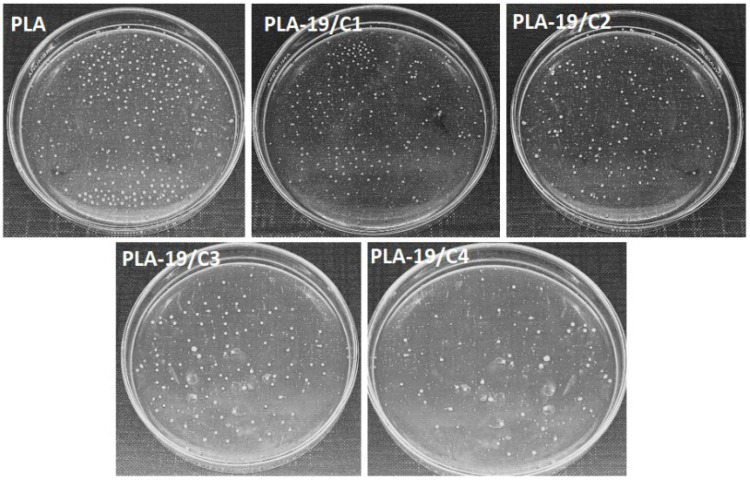
Quantitative measurement of antimicrobial activities of 19% PLA nanofibers with different CND concentrations against *S. aureus* bacteria.

The quantitative measurements of the antimicrobial activity against the *S. aureus* are shown in Figure 9; the antimicrobial efficiency increased from 16.7 % to 77.3% as the concentrations of the CNDs increased from 1% to 4%. It was also observed that at constant concentrations of CNDs, the antimicrobial activity was higher against the *S. aures* as compared to the *E. coli*. This can be explained by the fact that the cell wall of the Gram-negative bacterium, *E. coli*, is sandwiched and protected by two phospholipid bilayers; thus, the outer lipid layer resists the reactive oxygen species, resulting in less antimicrobial activity [25,26].

### 4.4. FTIR

**Figure 10:**
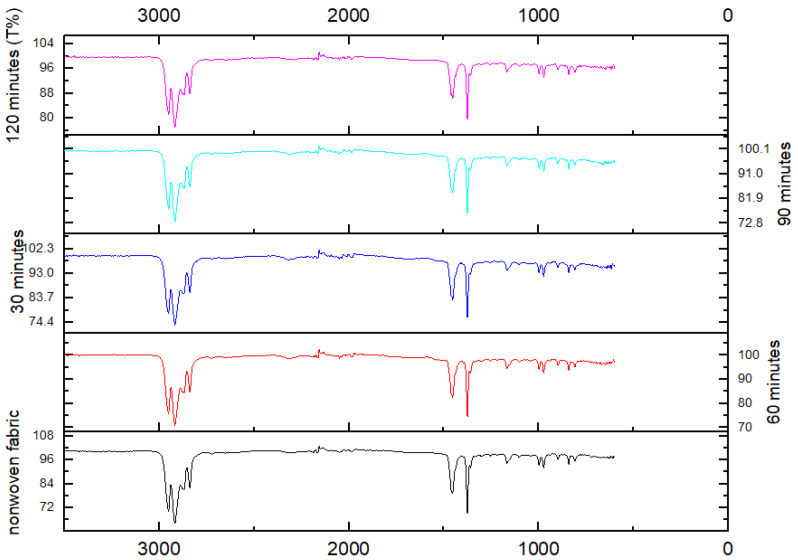
FTIR spectra of PLA-19/C4 samples at different electrospinning times.

The PLA/CNDs nanofibers were electrospun on a PLA nonwoven fabric. After 24 h of electrospinning, we performed the FTIR analysis. Due to the reduced electrospinning time, the weight percentage of the nanofibers collected on the nonwoven PLA sheet was very low. Due to the stacking of the FTIR data of all the samples and comparing them to the nonwoven PLA-collecting sheet, no differences were present in the samples, as shown in Figure 10. This suggests that no additional functional groups were formed. Furthermore, as we dissolved the PLA/CNDs in the DMF solution, the FTIR spectra of all the samples did not show any additional functional groups representing the DMF, leading to the conclusion there was no residual DMF in the nanofiber structure.

### 4.5. TGA

**Figure 11:**
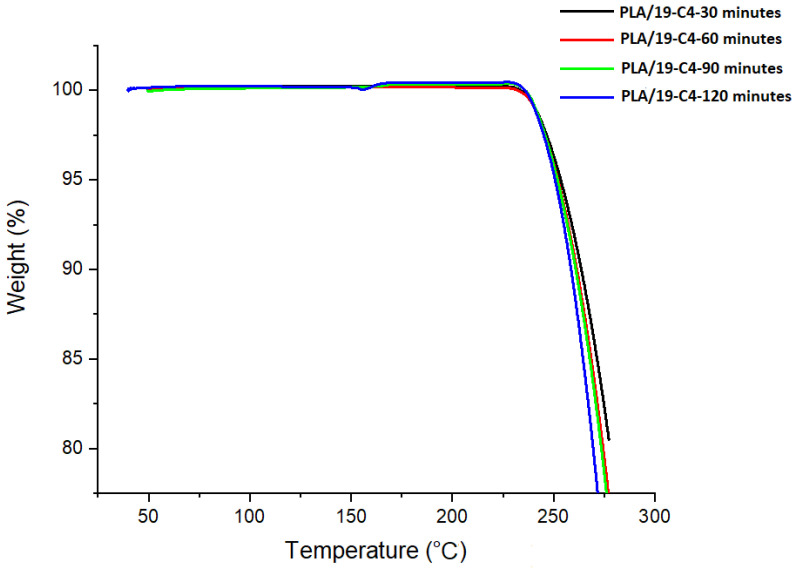
TGA analysis of PLA-19/C4 electrospun fibers.

The as-collected nanofibers on the nonwoven sheet were then subjected to thermogravimetric analysis (TGA), which is an analytical technique used to determine the thermal stability of materials and its fraction of volatile components by monitoring the weight change that occurs as a sample is heated. The samples were subjected to TGA analysis after 24 h of formation. The TGA curve in Figure 11 shows that all the samples had the same reaction to heat. A straight line until 240 °C was observed, after which decomposition/weight loss started at around 250 °C. No prominent curves or spikes were observed, which suggests that after 24 h of the formation of nanofibers on the nonwoven sheet, there was no residual DMF in the sheet. The residual DMF was the main concern with these nanofibers, as its intended application is in medical devices.

### 4.6. Particle-Filtration Efficiency and Differential Pressure

As the main attribute of a medical face mask is its filtration efficiency, we conducted the particle-filtration-efficiency and differential-pressure tests, which are the main requirements according to ASTM-F2100-20. We performed the electrospinning of PLA-19/C4, as it showed the highest antimicrobial efficiency amongst other concentrations. This PLA-19/C4 was then electrospun on the nonwoven sheet for different time periods 30, 60, 90, and 120 min.

Table 2 shows that with the increase in the electrospinning time, the particle-filtration efficiency was increased from 80% to 100%, which was due to the higher nanofiber density that collected on the nonwoven sheet when the electrospinning time was increased. As more nanofibers were collected on the surface of the nonwoven sheet, this might have affected the breathability of the face masks, so we also checked the differential pressure, and we observed that for the PLA-19/C4 electrospun at 30 min and 120 min, the values of the differential pressure were outside the range of the instrument’s minimum and maximum values. This means that the differential pressure was too low for the PLA-19/C4 electrospun for 30 min and that the differential pressure was too high for the PLA-19/C4 with the 120-min electrospinning time. For the samples at 60 min and 90 min, the differential pressure was in the range of 33.6 and 39.0 Pa/cm^2^, respectively. These values are perfect for Type I medical face masks, as mentioned in ASTM-F2100-20.

With respect to the different electrospinning times, the weight add-on to the rotating-drum collector is shown in Table 2. The nanofiber grammage was very low (0.447 mg/cm^2^) compared to the meltblown sheet (1.77 mg/cm^2^), which is normally used as a filtration layer in medical face masks. Thus, by using sustainable, biodegradable PLA/CND nanofibers as a filtration layer, we saved almost 75% of the PLA material’s cost without compromising the barrier performance of the medical face mask.

## 5. Conclusions

This study concludes that the use of 19% PLA in DMFs produced nanofibers with perfect morphology. The other concentrations of 12, 15, and 17% produced nanofibers but with more beads, and we also found that with the increase in concentration from 12% to 19%, the bead formation decreased and finer, smoother nanofibers were formed. As carbon nanodots are reported to have photocatalytic properties, we introduced different proportions of CNDs in PLA-19 nanofibers and found that with the increase in the concentrations of the CNDs from 1 to 4%, the antibacterial efficiency was also increased. In addition, we found that when the PLA/19-C4 was electrospun for 90 min on a spunbond sheet, it formed a perfect layered assembly, with a good PFE, of 96.5%, and a differential pressure of 39 Pa/cm^2^. We suggest that the use of this nanofiber layer sandwiched between spunbond layers makes a perfect filter layer that can be used for Type I medical face masks. The PLA is also a biobased and conditionally degradable polymer, which can also address the waste problems incurred by medical face masks made from non-sustainable sources.

## Figures and Tables

**Table 1:** Design of experiment, viscosity, and conductivity of different solutions of PLA in DMF.

* Sample Number	Concentration of PLA (%, *w*/*v*)	Concentration of CNDs (%)	Viscosity (mPa·s)	Conductivity (µS·cm)
PLA-12	12	–	177	4
PLA-15	15	–	230	6
PLA-17	17	–	285	8
PLA-17/C1	17	1	288	450
PLA-17/C2	17	2	291	666
PLA-17/C3	17	3	296	1020
PLA-17/C4	17	4	305	1260
PLA-19	19	–	335	12
PLA-19/C1	19	1	339	465
PLA-19/C2	19	2	345	702
PLA-19/C3	19	3	351	1065
PLA-19/C4	19	4	358	1290

* The number after PLA donates the percentage of PLA, and the number after C donates the percentage of CNDS in solution.

**Table 2:** Particle-filtration efficiency and differential pressure of PLA-19/C4 fibers on nonwoven sheet.

Sample	% PLA	% CNDs	Electrospinning Time (min)	Weight Add-On (mg/cm^2^)	Particle-Filtration Efficiency (%)	Differential Pressure (Pa/cm^2^)
PLA-19/C4	19	4%	30	0.149	80.0	Over range
			60	0.298	85.3	33.6
			90	0.447	96.5	39.0
			120	0.596	100	Over range
Meltblown sheet				1.78	95.	38.5

## Data Availability

The data of this study can be made available from the corresponding author (M.U.M.) upon reasonable request.
